# Supplementary material for: Impact of asymptomatic Plasmodium falciparum infection on the risk of subsequent symptomatic malaria in a longitudinal cohort in Kenya
Source: eLife. 2021 Jul 23;10:e68812. doi: 10.7554/eLife.68812 (PMC8337072; doi:10.7554/eLife.68812)
Supplement: Supplementary file 1. [file elife-68812-supp1.docx]

**Comparison of time-varying exposure coding approaches**

| **Exposure coding method** | **Description** | **Rationale for including/excluding** |
| --- | --- | --- |
| **Intention-to-treat** | Take exposure status at baseline and apply it over the full follow-up period | Can misclassify person-time if exposure frequently changes over time, as happens with the exposure in our study; this was commonly done in previous time to symptomatic malaria studies (Henning et al., 2004; Le Port et al., 2008; Liljander et al., 2011; Males et al., 2008; Njama-Meya et al., 2004; Nsobya et al., 2004; Portugal et al., 2017; Sonden et al., 2015; Wamae et al., 2018) |
| **Allow participants to change exposure group over follow-up** | Exposure is reassessed for participants over time and summarized as the number of months exposed | Can have issues with left truncation bias for exposures that began before the study, as occurred in our study where participants could have been infected with asymptomatic malaria at baseline |
| **Ever-never approach** | Classify participant as exposed if were ever exposed during follow-up period | Many issues with misclassification and “look-back” bias; Buchwald *et al.* (Buchwald et al., 2019) did a modified version of this where participants were classified as unexposed until an asymptomatic infection occurred then classified as exposed for the remaining period afterward |
| **Hernán *et al.* multiple month method**(Hernán et al., 2005) | Modified version of intention-to-treat where each month was treated as a baseline for follow-up; The exposure status of each monthly visit was applied to the subsequent follow-up period | Allows exposure to change over time with more precision than the typical intention-to-treat approach; produces effect estimate that is predictive of future risk regardless of prior exposure so not prone to left truncation bias; some misclassification bias still possible but less than alternative methods |

**SUPPLEMENTAL REFERENCES**

1. Njama-Meya D, Kamya MR, Dorsey G. Asymptomatic parasitaemia as a risk factor for symptomatic malaria in a cohort of Ugandan children. *Trop Med Int Heal*. 2004;9(8):862-868. doi:10.1111/j.1365-3156.2004.01277.x

2. Le Port A, Cot M, Etard JF, Gaye O, Migot-Nabias F, Garcia A. Relation between Plasmodium falciparum asymptomatic infection and malaria attacks in a cohort of Senegalese children. *Malar J*. 2008;7. doi:10.1186/1475-2875-7-193

3. Portugal S, Tran TM, Ongoiba A, Bathily A, Li S, Doumbo S, Skinner J, Doumtabe D, Kone Y, Sangala J, Jain A, Davies DH, Hung C, Liang L, Ricklefs S, Homann MV, Felgner PL, Porcella SF, Färnert A, Doumbo OK, Kayentao K, Greenwood BM, Traore B, Crompton PD. Treatment of chronic asymptomatic plasmodium falciparum infection does not increase the risk of clinical malaria upon reinfection. *Clin Infect Dis*. 2017;64(5):645-653. doi:10.1093/cid/ciw849

4. Sonden K, Doumbo S, Hammar U, Vafa Homann M, Ongoiba A, Traord B, Bottai M, Crompton PD, Färnert A. Asymptomatic Multiclonal Plasmodium falciparum Infections Carried Through the Dry Season Predict Protection Against Subsequent Clinical Malaria. *J Infect Dis*. 2015;212(4):608-616. doi:10.1093/infdis/jiv088

5. Henning L, Schellenberg D, Smith T, Henning D, Alonso P, Tanner M, Mshinda H, Beck HP, Felger I. A prospective study of Plasmodium falciparum multiplicity of infection and morbidity in Tanzanian children. *Trans R Soc Trop Med Hyg*. 2004;98(12):687-694. doi:10.1016/j.trstmh.2004.03.010

6. Liljander A, Bejon P, Mwacharo J, Kai O, Ogada E, Peshu N, Marsh K, Färnert A. Clearance of asymptomatic P. falciparum infections interacts with the number of clones to predict the risk of subsequent malaria in Kenyan children. *PLoS One*. 2011;6(2). doi:10.1371/journal.pone.0016940

7. Wamae K, Wambua J, Nyangweso G, Mwambingu G, Osier F, Ndung’u F, Bejon P, Ochola-Oyier LI. Transmission and Age Impact the Risk of Developing Febrile Malaria in Children with Asymptomatic Plasmodium falciparum Parasitemia. *J Infect Dis*. Published online 2018. doi:10.1093/infdis/jiy591

8. Nsobya SL, Parikh S, Kironde F, Lubega G, Kamya MR, Rosenthal PJ, Dorsey G. Molecular Evaluation of the Natural History of Asymptomatic Parasitemia in Ugandan Children. *J Infect Dis*. 2004;189(12):2220-2226. doi:10.1086/421281

9. Males S, Gaye O, Garcia A. Long-Term Asymptomatic Carriage of Plasmodium falciparum Protects from Malaria Attacks: a Prospective Study among Senegalese Children. *Clin Infect Dis*. 2008;46(4):516-522. doi:10.1086/526529

10. Buchwald AG, Sixpence A, Chimenya M, Damson M, Sorkin JD, Wilson ML, Seydel K, Hochman S, Mathanga DP, Taylor TE, Laufer MK. Clinical Implications of Asymptomatic Plasmodium falciparum Infections in Malawi. *Clin Infect Dis*. 2019;21201(1):106-112. doi:10.1093/cid/ciy427

11. Hernán MA, Robins JM, García Rodríguez LA. Discussion on “statistical issues arising in the Women’s Health Initiative.” *Biometrics*. 2005;61(4):922-930. doi:10.1111/j.0006-341X.2005.454_6.x
