# Supplementary material for: Impact of asymptomatic Plasmodium falciparum infection on the risk of subsequent symptomatic malaria in a longitudinal cohort in Kenya
Source: eLife. 2021 Jul 23;10:e68812. doi: 10.7554/eLife.68812 (PMC8337072; doi:10.7554/eLife.68812)
Supplement: Supplementary file 2. [file elife-68812-supp2.docx]

**Covariate distribution across symptomatic events: secondary permissive case definition**

|  | **Total person-months***  (N, %) | **Person-months ending in symptomatic infections****  (N, %) | **Median time to symptoms**  (days, IQR) | ***P*-value** |
| --- | --- | --- | --- | --- |
| **Main exposure** |  |  |  | <0.001^a^ |
| No infection | 3537 (65.8) | 2122 (67.0) | 190 (80, 333) |  |
| Asymptomatic infection | 1837 (34.2) | 1044 (33.0) | 137 (41, 308) |  |
| **Age** |  |  |  | 1.000^b^ |
| < 5 years | 806 (15.0) | 419 (13.2) | 182 (57, 345) |  |
| 5-15 years | 2280 (42.4) | 1556 (49.1) | 174 (64, 337) |  |
| > 15 years | 2288 (42.6) | 1191 (37.6) | 169 (64, 310) |  |
| **Sex** |  |  |  | 0.133^a^ |
| Male | 2374 (44.2) | 1468 (46.4) | 186 (67, 349) |  |
| Female | 3000 (55.8) | 1698 (53.6) | 163 (61, 308) |  |
| **Regular bed net usage^#^** |  |  |  | 1.000^a^ |
| No | 1445 (26.9) | 876 (27.7) | 179 (67, 342) |  |
| Yes | 3929 (73.1) | 2290 (72.3) | 171 (63, 320) |  |
| **Village** |  |  |  | 1.000^b^ |
| Kinesamo | 1846 (34.4) | 1073 (33.9) | 181 (65, 319) |  |
| Maruti | 1669 (31.1) | 1013 (32.0) | 159 (60, 314) |  |
| Sitabicha | 1859 (34.6) | 1080 (34.1) | 182 (68, 348) |  |
|  |  |  |  |  |

Abbreviations: IQR, interquartile range

^#^Regular bed net usage was defined as a person averaging > 5 nights a week sleeping under a bed net.

*Total person-months indicates the total number of monthly follow-up visits ending in a symptomatic infection or censoring.

**Symptomatic infections were defined using the secondary permissive case definition where a participant was *P. falciparum*-positive by qPCR as well as had at least one symptom consistent with malaria during a sick visit.

^a^ Wilcoxon Rank Sum test with continuity correction and Bonferroni correction for repeated measures.

^b^ Kruskal-Wallis test with Bonferroni correction for repeated measures.
