# Supplementary material for: Impact of asymptomatic Plasmodium falciparum infection on the risk of subsequent symptomatic malaria in a longitudinal cohort in Kenya
Source: eLife. 2021 Jul 23;10:e68812. doi: 10.7554/eLife.68812 (PMC8337072; doi:10.7554/eLife.68812)
Supplement: Supplementary file 3. [file elife-68812-supp3.docx]

**Covariate distribution across symptomatic events: secondary stringent case definition**

|  | **Total person-months visits***  (N, %) | **Person-months ending in symptomatic infections****  (N, %) | **Median time to symptoms**  (days, IQR) | ***P*-value** |
| --- | --- | --- | --- | --- |
| **Main exposure** |  |  |  | 0.018^a^ |
| No infection | 3532 (65.7) | 1311 (65.6) | 232 (104, 403) |  |
| Asymptomatic infection | 1848 (34.3) | 687 (34.4) | 203 (52, 429) |  |
| **Age** |  |  |  | 0.106^b^ |
| < 5 years | 812 (15.1) | 329 (16.5) | 226 (82, 435) |  |
| 5-15 years | 2286 (42.5) | 1161 (58.1) | 209 (78, 389) |  |
| > 15 years | 2282 (42.4) | 508 (25.4) | 254 (103, 459) |  |
| **Sex** |  |  |  | 1.000^a^ |
| Male | 2355 (43.8) | 983 (49.2) | 236 (88, 436) |  |
| Female | 3025 (56.2) | 1015 (50.8) | 210 (82, 398) |  |
| **Regular bed net usage^#^** |  |  |  | 1.000^a^ |
| No | 1427 (26.5) | 645 (32.3) | 210 (84, 380) |  |
| Yes | 3953 (73.5) | 1353 (67.7) | 233 (86, 432) |  |
| **Village** |  |  |  | 0.032^b^ |
| Kinesamo | 1853 (34.4) | 695 (34.8) | 241 (94, 440) |  |
| Maruti | 1680 (31.2) | 643 (32.2) | 186 (66, 376) |  |
| Sitabicha | 1847 (34.3) | 660 (33.0) | 238 (91, 427) |  |
|  |  |  |  |  |

Abbreviations: IQR, interquartile range

^#^Regular bed net usage was defined as a person averaging > 5 nights a week sleeping under a bed net.

*Total person-months indicates the total number of monthly follow-up visits ending in a symptomatic infection or censoring.

**Symptomatic infections were defined using the secondary stringent case definition where a participant was *P. falciparum*-positive by both RDT and qPCR as well as had a self-reported fever during a sick visit.

^a^ Wilcoxon Rank Sum test with continuity correction and Bonferroni correction for repeated measures.

^b^ Kruskal-Wallis test with Bonferroni correction for repeated measures.
