# Supplementary material for: Impact of asymptomatic Plasmodium falciparum infection on the risk of subsequent symptomatic malaria in a longitudinal cohort in Kenya
Source: eLife. 2021 Jul 23;10:e68812. doi: 10.7554/eLife.68812 (PMC8337072; doi:10.7554/eLife.68812)
Supplement: Transparent reporting form [file elife-68812-transrepform.docx]

***eLife’s* transparent reporting form**

We encourage authors to provide detailed information *within their submission* to facilitate the interpretation and replication of experiments. Authors can upload supporting documentation to indicate the use of appropriate reporting guidelines for health-related research (see [EQUATOR Network](http://www.equator-network.org/%20)), life science research (see the [BioSharing Information Resource](https://biosharing.org/)), or the [ARRIVE guidelines](http://www.plosbiology.org/article/info:doi/10.1371/journal.pbio.1000412) for reporting work involving animal research. Where applicable, authors should refer to any relevant reporting standards documents in this form.

If you have any questions, please consult our Journal Policies and/or contact us: [editorial@elifesciences.org](mailto:editorial@elifesciences.org).

**Sample-size estimation**

- You should state whether an appropriate sample size was computed when the study was being designed
- You should state the statistical method of sample size computation and any required assumptions
- If no explicit power analysis was used, you should describe how you decided what sample (replicate) size (number) to use

Please outline where this information can be found within the submission (e.g., sections or figure legends), or explain why this information doesn’t apply to your submission:

The study sample size was pre-determined based on enrolling up to 36 households in a research grant and no explicit power analysis was conducted. Two households moved out of the study period during follow-up and were replaced by two new households, giving a total of 38 households sampled. The number of households chosen to sample was based on a prior cross-sectional study in the area. We ended up enrolling more participants than anticipated in the grant (N=268 vs. 190) based on more participants residing within households than anticipated. The study sample size is described in the first paragraph of the methods section (page 4) and of the results section (page 8) of the manuscript.

**Replicates**

- You should report how often each experiment was performed
- You should include a definition of biological versus technical replication
- The data obtained should be provided and sufficient information should be provided to indicate the number of independent biological and/or technical replicates
- If you encountered any outliers, you should describe how these were handled
- Criteria for exclusion/inclusion of data should be clearly stated
- High-throughput sequence data should be uploaded before submission, with a private link for reviewers provided (these are available from both GEO and ArrayExpress)

Please outline where this information can be found within the submission (e.g., sections or figure legends), or explain why this information doesn’t apply to your submission:

The dried blood spot processing and qPCR conducted to detect *P. falciparum* is described in the materials and methods section on page 4 under “Study population, sample collection, and sample processing.” Specific details on how the main exposure and outcome, lost to follow-up, and censoring were conducted are provided on page 5. Sensitivity analyses were performed using additional case definitions for symptomatic malaria, different modelling approaches, and different inclusion/exclusion criteria for possibly pre-symptomatic infections. Results of sensitivity analyses are largely recorded in the supplemental information.

**Statistical reporting**

- Statistical analysis methods should be described and justified
- Raw data should be presented in figures whenever informative to do so (typically when N per group is less than 10)
- For each experiment, you should identify the statistical tests used, exact values of N, definitions of center, methods of multiple test correction, and dispersion and precision measures (e.g., mean, median, SD, SEM, confidence intervals; and, for the major substantive results, a measure of effect size (e.g., Pearson's r, Cohen's d)
- Report exact p-values wherever possible alongside the summary statistics and 95% confidence intervals. These should be reported for all key questions and not only when the p-value is less than 0.05.

Please outline where this information can be found within the submission (e.g., sections or figure legends), or explain why this information doesn’t apply to your submission:

Statistical methods details are provided on pages 5-7 of the manuscript. The results from these statistical approaches are reported on pages 8-10 of the manuscript. Exact values of N used in statistical tests are reported in table 1 of the manuscript with associated p-values. Hazard ratios produced by multi-level cox proportional hazards models are reported with their corresponding 95% confidence intervals in tables 2 and 3. Additional results are provided in the results main text as well as the supplement.

(For large datasets, or papers with a very large number of statistical tests, you may upload a single table file with tests, Ns, etc., with reference to sections in the manuscript.)

**Group allocation**

- Indicate how samples were allocated into experimental groups (in the case of clinical studies, please specify allocation to treatment method); if randomization was used, please also state if restricted randomization was applied
- Indicate if masking was used during group allocation, data collection and/or data analysis

Please outline where this information can be found within the submission (e.g., sections or figure legends), or explain why this information doesn’t apply to your submission:

The study was a longitudinal observational cohort so samples were not randomized to groups. Demographic questionnaires were collected during follow-up and responses used to determine covariate groupings.

**Additional data files (“source data”)**

- We encourage you to upload relevant additional data files, such as numerical data that are represented as a graph in a figure, or as a summary table
- Where provided, these should be in the most useful format, and they can be uploaded as “Source data” files linked to a main figure or table
- Include model definition files including the full list of parameters used
- Include code used for data analysis (e.g., R, MatLab)
- Avoid stating that data files are “available upon request”

Please indicate the figures or tables for which source data files have been provided:

All R code used for data analysis is provided on Github:  [https://github.com/duke-malaria-collaboratory/time_to_symptomatic_malaria](https://urldefense.com/v3/__https:/github.com/duke-malaria-collaboratory/time_to_symptomatic_malaria__;!!OToaGQ!-Nd1N7MQYUrvXfq6HlkFdjX-fa-ZLmgG2wnEQF4128cFUGUqlRv0hF-VWHOQgC6I5Jk$). Following our IRB protocol and to ensure protection of our study participants’, we cannot provide the raw, longitudinal line level data files used for analyses, figures, and tables .
